# Opinion of surveyed nurses on transplantation and reasons for negative public attitudes toward organ donation

**DOI:** 10.3389/frtra.2023.1193680

**Published:** 2023-05-09

**Authors:** Bożena Majchrowicz, Katarzyna Tomaszewska, Beata Guzak

**Affiliations:** ^1^ Department of Nursing, Institute of Health Protection, State Academy of Applied Sciences, Przemyśl, Poland; ^2^ Department of Nursing, Institute of Health Protection, The Bronislaw Markiewicz State Higher School of Technology and Economics, Jarosław, Poland; ^3^ Center of Postgraduate Education for Nurses and Midwives, Warsaw, Poland

**Keywords:** transplant nurse, society, knowledge, education, attitudes

## Abstract

Organ transplantation saves thousands of lives every year. Despite growing awareness of transplantation, the issue of obtaining organs for transplantation has been controversial for years. Hundreds of people are waiting in lines all the time for transplantation, for whom it is the only hope for a cure. One extremely important factor contributing to the low number of transplants is the low number of organ donations from deceased donors. Nurses are considered key facilitators of the organ procurement and transplantation process. Their knowledge of and attitudes toward organ donation can influence public opinion, as well as the decisions of their families to donate the organs of the deceased. The purpose of our study was to determine the opinions of surveyed nurses about transplantation and the reasons for negative public attitudes toward organ donation. The survey included 793 nurses employed in surgical wards across Poland with varying levels of job seniority. The survey was conducted between September and October 2022. The research tool was a survey questionnaire, consisting of three parts: socio-demographic data, questions assessing the respondents' knowledge of behavior about transplantation, and a non-standardized tool to measure respondents' emotional and motivational attitudes toward transplantation. Participation in the survey was anonymous and voluntary. The statistical analysis for independence of variables used the *χ*^2^ test. On the other hand, coefficients based on the Phi and Cramer's V test, as well as Kruskal Wallis non-parametric tests for assessing differences (for more than 2 samples) were used to determine the strength of the relationship. During these analyses, in addition to standard statistical significance, the corresponding “*p*” values were calculated using the Monte Carlo method. According to the nurses surveyed, transplantation is a life-saving procedure. A significant proportion of the nurses (85.6%) believe that there are too few donors in Poland. According to 41.8% of the respondents, this is due to the fear of misdiagnosis of death, for 23.4% it is incompatible with their worldview or religion and 31.8% believe it is due to the belief in the inviolability of the human body after death. The survey showed that, in the nurses' opinion, the reason for objections to organ transplantation is the deficit in public's knowledge of cell, tissue and organ donation from both living and dead donors. Therefore it is extremely important to conduct educational campaigns in this regard.

## 1. Introduction

The problem of cell, tissue and organ transplantation, due to the social and legal importance of the issue, as well as the many ethical dilemmas associated with organ procurement from deceased donors on the basis of so-called presumed consent, has become the subject of numerous acts of international law. Guidelines on the subject of organ donation for transplantation were also issued by the World Health Organization (WHO) in 1991, all with the aim of creating a framework system of orderly and socially acceptable regulations for obtaining and transplanting human organs to save health and life. According to WHO recommendations, it is desirable that organs for transplantation be obtained from the dead (1, 2). Organ transplantation saves thousands of lives every year. Despite growing awareness of transplantation, there are still hundreds of people waiting in lines for transplantation, for whom it is the only hope. Some of them die while waiting in line. One extremely important factor that contributes to the insufficient number of transplants is the low number of organ donations from deceased donors (3–5).

Nurses are considered key facilitators of the organ procurement and transplantation process by obtaining family consent for donation (6–8). Their knowledge of and attitudes toward organ donation can influence public opinion, as well as decisions of their family members to donate the deceased's organs (9, 10). Nurses are important medical personnel caring for potential organ donors, and their low level of knowledge and attitude toward organ donation can exacerbate the lack of organ availability for transplantation (11, 12). Therefore it is important to increase nurses' knowledge of the principles of organ donation (13, 14) and to understand their views and experiences in this regard (15, 16). Knowledge and attitudes toward organ donation are the most important determinants of willingness to donate an organ (17–19). The aim of our study was to determine the opinions of the nurses surveyed about transplantation and the reasons for negative public attitudes toward organ donation.

## 2. Material and methods

### 2.1. Research design

In the present study, a survey was conducted among working nurses employed in surgical wards in Polish public health sector who were providing work during the survey. A group of female surgical nurses was chosen for this study because in Poland they work in surgical and trauma wards (78.0% of respondents) as well as emergency departments (22.0% of respondents), the places where patients who could become potential donors are most often admitted. The survey was conducted between September and October 2022. The survey-based, descriptive cross-sectional study was conducted to assess nurses' knowledge, attitudes and awareness of organ donation and to learn about the surveyed nurses' opinions about organ transplantation and the reasons for society's negative attitudes toward organ donation.

### 2.2. Research tools

The first part of the research tool was a self-administered survey questionnaire containing 9 questions on sociodemographic data and 14 questions designed to assess the respondents' level of knowledge about transplantation. The knowledge component was built based on a two-point scale—if the respondent provided a correct answer, 1 point was given, and if the answer was wrong, no points were given. In addition, the next part of the questionnaire used a non-standardized tool to measure respondents' emotional and motivational attitudes toward transplantation, developed by M. Perkowska, which was used with the author's permission. The scale contained 20 statements on the basis of which it was possible to determine the emotional-motivational attitude to organ procurement for transplantation. Respondents could answer each statement by choosing one of five possibilities, where 5 – completely agree; 4 – rather agree; 3 – have no opinion (difficult to decide); 2 – rather disagree; 1 – completely disagree.

### 2.3. Participants

The study group consisted of 793 nurses employed in surgical wards of hospitals in Poland, recruited by non-probabilistic sampling. Each respondent independently and voluntarily completed a survey questionnaire and gave written consent to participate in the study, and each respondent was provided with information about the processing of respondents' personal data. The consents and survey questionnaires are in the possession of the authors of this paper. Initially, 1000 questionnaires were distributed, 793 were accepted and correctly completed, which was 79.3%. The criterion for inclusion in the study was current employment in the surgical ward and consent to participate in the study. The questionnaires were left in the nursing questionnaires and after completion were collected by the study authors.

### 2.4. Statistical analysis

In the analysis of the collected material, descriptive statistics were used to describe the most important information about the variables analyzed in the study and the study group. The basic test that was used in the statistical analyses is the *χ*^2^ test for independence of variables. It was mainly used for questions built on nominal scales. Coefficients based on the aforementioned test, Phi and Cramer's V, were used to determine the strength of the relationship. The Phi measure also indicates the direction of the relationship (positive or negative). When the variables were ordinal, Kendall's Tau-*c* coefficients for tables with different numbers of columns and rows were used, respectively. Correlations between ordinal or quantitative variables (when the conditions for using parametric tests were not met) were made using Spearman's rho coefficient, which indicates the intensity of the relationship and its direction—positive or negative. The resulting value ranges from −1 to 1, with (−1) indicating a perfect negative correlation and (1) a perfect positive correlation. Underneath each crosstab was the value of the coefficient and statistical significance “*p*”. In addition, the “*p*” value is calculated using the Monte Carlo method, which is also indicated by a letter. The analysis was performed using the IBM SPSS 26.0 package (IBM, New York City, NY, United States) with the Exact Tests module. All relationships, correlations and differences were statistically significant when *p* ≤ 0.05.

### 2.5. Ethical procedures

The participation of nurses in the study was voluntary and anonymous. The study was conducted in accordance with the ethical standards set forth in the Declaration of Helsinki (64th WmA General Assembly, Fortaleza, Brazil, October 2013) and in accordance with Polish legal regulations. The application was approved by the Bioethics Committee of the State Academy of Applied Sciences in Przemyśl (KBPWSW No. 06/2022).

## 3. Results

The survey was designed to provide the opinions of surveyed nurses employed in surgical wards about transplantation and the reasons for negative public attitudes toward organ donation. A total of 793 nurses were surveyed. The characteristics of the surveyed group are shown in Table 1.

**Table 1:** Characteristics of the study group.

Variable		Frequency (*n = 793*)	
Gender	Female	763	96.3%
	Male	29	3.7%
Job seniority (years)	<5	297	37.5%
	6–15	248	31.3%
	16–25	115	14.5%
	>25	132	16.7%
Education*	Secondary	36	4.5%
	Medical school	27	3.4%
	Bachelor's degree	159	20.1%
	Master's degree	570	72.0%
Marital status	Married	499	63.0%
	Single	134	16.9%
	Divorced	64	8.1%
	Separated	3	0.4%
	Widowed	11	1.4%
	In a free relationship	45	5.7%
	Single	36	4.5%
Religion	Roman Catholic	727	91.8%
	Jehovah's Witness	1	0.1%
	Orthodox	1	0.1%
	Atheist	45	5.7%
	Other	18	2.3%
Number of children	0	273	34.5%
	1	229	28.9%
	2	232	29.3%
	3+	58	7.3%

*Until 2000, in Poland, pre-graduate education for nurses took place in a 5-year medical high school after graduating from an 8th grade elementary school or a 2/2.5-year medical college after graduating from high school. Since 2000, pre-graduate education for nurses and midwives has been conducted in a two-degree university system: upon completion of the first degree, which lasts 3 years, nurses are awarded a Bachelor's degree in nursing; upon completion of the second degree, which lasts 2 years, they are awarded a Master's degree in nursing.

### 3.1. Results of the author's survey questionnaire

According to 98.7% of surveyed nurses, transplantation is a life-saving procedure. Repeatedly the only way to prolong a patient's life is to transplant an organ. In Poland, the removal of organs from a deceased person is conditioned by law and there is implied consent (78.3%) and, according to 17.0% of respondents, the consent of the family. 1.5% of respondents believe that legally it is not possible to take organs from a deceased person, while 3.2% had no knowledge in this regard. A large part of the nurses surveyed (85.6%) believe that there are too few donors in Poland. According to 41.8% of respondents, this is due to the fear of incorrect diagnosis of death, for 23.4% it is incompatible with worldview or religion and 31.8% believe it is due to the belief in the inviolability of the human body after death.

87.0% of respondents know the correct definition of brain death. The relationship between age and the question “do you know what brain death is” was found to be statistically significant, but the strength of the relationship is not significant. The educational level of the respondents statistically significantly differentiates the answers of the respondents (*p* < 0.001, Monte Carlo *p* < 0.001, *χ*^2^ = 33.60, df-2, Cramer's *V* = 0.29). It has also been shown that higher levels of knowledge about brain death are associated with greater support for transplantation, and this is most evident in the case of donating one's own organs. Although the value of the correlation coefficient is quite weak Kendall's Tau-*b* = −0.20 it is significantly different from 0 (*p* < 0.001, Monte Carlo *p* < 0.001).

When asked what the respondents' attitude is toward donating organs such as lungs, heart, cornea, kidneys, liver after death to a stranger, 80.1% of nurses declared strong support, rather support 13.3% of respondents, hard to say was marked by 4.4%, rather not support was indicated by 0.4% while strongly not support was indicated by 1.8% of respondents. The results of the question on organ donation after the death of a loved one were similar to the above.

According to 93.4% of respondents, promoting knowledge among the public about informed organ donation is very necessary, since in the opinion of 90.3% of respondents, it is having knowledge about transplantation that can make the difference in an individual's decision to donate organs. 62.2% of the respondents have heard of public campaigns on transplantation. Only 34.5% of the surveyed nurses of their professional work took any action (e.g., talking to colleagues, patients) to promote the idea of transplantation. According to 90.3% of respondents, having knowledge about transplantation can make a big difference in an individual's decision to donate organs. According to the nurses surveyed, knowledge about transplantation should be provided by schools (23%), universities (21.4%), the Church and religious associations (12.9%), the media (29.0%), and the medical community (13.7%).

The value of the coefficient of the relationship between the place of work and the ratio of support for donating organs after death that belong to the respondents is statistically significant, but the strength of the relationship was found to be not significant. Analyzing the results in the table, there are no clear differences in support considering the place of work (Cramer's *V* = 0.061, *χ*^2^ = 2.847, Monte Carlo *p* = 0.598).

The value of the correlation coefficient between education and the ratio of support for donating organs after death that belong to a loved one is statistically significant, but the strength of the relationship was not significant. Analyzing the results in the table, there is no clear trend of support considering education (Kendall's tau-*c* = 0.054, Monte Carlo *p* = 0.002).

The value of the correlation coefficient between seniority and the ratio of support for donating organs after death that belong to a stranger is statistically significant, but the strength of the relationship turned out to be practically zero. Analyzing the results in the table, there is no clear trend of support for donating organs after one's own death due to the seniority of the respondents (Table 2).

**Table 2:** Support for the idea of transplantation according to the seniority of respondents.

Variables			Job seniority (years)				Total
			>5	6–15	16–25	>25	
What is your attitude to support the donation of organs after death that belong to yourself	I strongly support	Frequency (*n*)	237	202	79	96	614
		Percentage (%)	81.2	86.0	76.7	82.8	82.3
	I rather support	Frequency (*n*)	35	19	15	14	83
		Percentage (%)	12.0	8.1	14.6	12.1	11.1
	It is difficult to say	Frequency (*n*)	14	11	6	4	35
		Percentage (%)	4.8	4.7	5.8	3.4	4.7
	I rather do not support	Frequency (*n*)	1	1	1	1	4
		Percentage (%)	0.3	0.4	1.0	0.9	0.5
	I definitely do not support	Frequency (*n*)	5	2	2	1	10
		Percentage (%)	1.7	0.9	1.9	0.9	1.3
Total		Frequency (*n*)	292	235	103	116	746
		Percentage (%)	100.0	100.0	100.0	100.0	100.0
Kendall's tau-*c*	−0.001	0.021	−0.046	0.963	0.964		
Coefficient	Value	Standard error	Approximate *T*	*p*	Monte Carlo *p*	** **	
Cramer's *V*	0.055	6.876	12	0.866	0.879		
Coefficient	Value	*χ* ^2^	*df*	*p*	Monte Carlo p	** **	

### 3.2. Results of a scale to assess emotional and motivational attitudes toward transplantation

The results of the statements were ordered from the highest degree of agreement to the lowest degree of agreement (Table 3).

**Table 3:** Feelings and motivations of respondents towards transplantation (*n* = 793).

Statements	Mean	Median	Standard deviation
Transplantation is one of the methods of treatment.	4.55	5.00	±0.798
Donating one's own cells, tissues, organs is a beautiful gesture/gift to another human being.	4.54	5.00	±0.883
When human life or health is at risk, it is the doctor's duty to save it at all costs.	4.50	5.00	±0.855
Conducting educational campaigns in the field of transplantology helps people make decisions about organ donation.	4.40	5.00	±0.914
The dynamic development of transplantology is admirable and commendable.	4.24	5.00	±0.968
Donating one's own cells, tissues, organs is a moral duty of every person.	3.45	3.00	±1.135
Transplantation of organs to people addicted to psychoactive substances should be banned.	3.22	3.00	±1.214
Being a living donor involves great risks to life and health.	2.77	3.00	±1.202
Taking organs from animals is harming and exploiting them.	2.73	3.00	±1.211
Taking organs from children should be banned, as someone else is making the decisions for them.	2.71	3.00	±1.269
Transplanting organs from animals is unethical, it harms human dignity.	2.59	3.00	±1.229
There is an underworld of transplantation in Poland.	2.53	3.00	±1.099
People who illegally obtain an organ for transplantation should not be punished.	2.46	3.00	±1.358
After transplantation, the recipient may reveal character traits, personalities, preferences characteristic of the donor.	2.15	2.00	±1.234
Transplantation is a morally controversial medical activity.	1.76	1.00	±1.140
Taking organs from a deceased person is desecration of a corpse.	1.54	1.00	±1.037
Accepting cells, tissues, organs from another person is a sin.	1.44	1.00	±0.988
Transplants are just medical experiments.	1.41	1.00	±0.923
A person who needs a transplant should bring the necessary tissues, cells, organs to the hospital at his own expense.	1.40	1.00	±0.913
Transplants should be banned.	1.33	1.00	±0.861

Education, job tenure and the number of children one has, for the most part, do not correlate in a statistically significant way with the statements placed on the scale. A small part of the values of the correlation coefficients, although statistically significant is practically characterized by a negligible strength of the relationship (Table 4).

**Table 4:** Results of questionnaire to assess level of acceptance of transplantation among respondents.

Variables			Education	Job seniority	Number of children
Spearman's rho	When human life or health is at risk, it is the doctor's duty to save it at all costs.	Correlation coefficient	−0.082*	0.099**	0.052
		Significance (two-tailed)	0.021	0.006	0.143
		Frequency (*n*)	784	783	787
	Transplantation is one of the methods of treatment.	Correlation coefficient	0.031	−0.037	−0.034
		Significance (two-tailed)	0.379	0.306	0.335
		Frequency (*n*)	785	784	788
	Transplants should be banned.	Correlation coefficient	−0.061	0.029	0.058
		Significance (two-tailed)	0.090	0.417	0.107
		Frequency (*n*)	784	783	787
	Taking organs from a dead person is desecration of a corpse.	Correlation coefficient	−0.081*	0.056	0.064
		Significance (two-tailed)	0.024	0.116	0.071
		Frequency (*n*)	782	781	785
	Accepting cells, tissues, organs from another person is a sin.	Correlation coefficient	−0.022	0.020	0.029
		Significance (two-tailed)	0.539	0.575	0.422
		Frequency (*n*)	780	779	783
	Transplants are just medical experiments.	Correlation coefficient	−0.039	0.013	0.035
		Significance (two-tailed)	0.278	0.710	0.327
		Frequency (*n*)	774	773	777
	Transplants are a morally controversial medical activity.	Correlation coefficient	−0.010	−0.030	0.008
		Significance (two-tailed)	0.772	0.410	0.815
		Frequency (*n*)	781	780	784
	A person who needs a transplant should bring the necessary tissues, cells, organs to the hospital at his own expense.	Correlation coefficient	−0.053	−0.032	0.006
		Significance (two-tailed)	0.140	0.375	0.869
		Frequency (*n*)	782	781	785
	After the transplant, the recipient may reveal character traits, personalities, preferences characteristic of the donor.	Correlation coefficient	−0.046	0.034	0.006
		Significance (two-tailed)	0.200	0.341	0.865
		Frequency (*n*)	783	782	786
	Donating one's own cells, tissues, organs is a beautiful gesture/gift to another person.	Correlation coefficient	0.009	0.037	0.058
		Significance (two-tailed)	0.791	0.300	0.106
		Frequency (*n*)	783	782	786
	Donating one's own cells, tissues. organs is a moral obligation of every human being.	Correlation coefficient	−0.078*	0.071*	0.013
		Significance (two-tailed)	0.030	0.048	0.716
		Frequency (*n*)	786	785	789
	Being a living donor involves great risks to life and health.	Correlation coefficient	−0.014	−0.032	−0.014
		Significance (two-tailed)	0.705	0.365	0.702
		Frequency (*n*)	779	778	782
	The dynamic development of transplantology is admirable and commendable.	Correlation coefficient	−0.036	0.008	−0.018
		Significance (two-tailed)	0.321	0.830	0.616
		Frequency (*n*)	764	764	767
	Transplantation of organs to people addicted to psychoactive substances should be banned.	Correlation coefficient	0.019	−0.050	−0.059
		Significance (two-tailed)	0.601	0.158	0.097
		Frequency (*n*)	784	783	787
	Conducting transplant education campaigns helps people make decisions about organ donation.	Correlation coefficient	−0.064	0.059	0.001
		Significance (two-tailed)	0.073	0.099	0.984
		Frequency (*n*)	782	781	785
	Transplanting organs from animals is unethical, harms human dignity.	Correlation coefficient	−0,064	0,042	0.025
		Significance (two-tailed)	0,072	0,237	0.481
		Frequency (*n*)	782	781	785
	People who illegally obtain an organ for transplantation should not be punished.	Correlation coefficient	−0.115**	0.098**	0.062
		Significance (two-tailed)	0.001	0.006	0.083
		Frequency (*n*)	786	785	789
	Getting organs from animals is harming and exploiting them.	Correlation coefficient	−0.051	0.099**	0.049
		Significance (two-tailed)	0.155	0.005	0.165
		Frequency (*n*)	788	787	791
	Getting organs from children should be banned, as someone else makes the decisions for them.	Correlation coefficient	−0.079*	0.053	0.090*
		Significance (two-tailed)	0.026	0.141	0.011
		Frequency (*n*)	787	786	790

^*^ Correlation significant at the 0.05 level (two-tailed).

^**^ Correlation significant at the 0.01 level (two-tailed).

The nurses surveyed (78.1%) believe that people have the right to choose and can make their own decisions about the purpose of their organs both during life and after death. The question asked to the nurses surveyed about the reasons for opposing organ donation was very important. According to 18.9% of the respondents, it is the deficit of public knowledge about the possibility of donating cells, tissues and organs from living donors and people who have been declared dead as a result of being pronounced dead, 11.6% said the influence of religion, 14.4% the reluctance to interfere with the body of the deceased. For 14.7%, the shock of a loved one's death, 7.0% considered the difficulty of making a decision, 5.2% lack of knowledge of the deceased person's will, 1.6% fear of doctors' dishonesty and for 4.4% concerns about the correctness of the death diagnosis.

These responses prove that there is a great need to expand the knowledge among our profession about transplantation, which will allow us to conduct effective education of the whole society.

## 4. Discussion

The study presents the opinions of the nurses surveyed about transplantation and the reasons for society's negative attitude toward organ donation. The author's study showed that, according to 98.7% of the respondents, transplantation is a life-saving procedure. Similarly, studies by other authors have found that nurses who would donate their organs have higher expertise, knowledge of the irreversibility of brain death and better attitudes toward organ donation (20, 21). Medical personnel support transplantation procedures because of their high awareness of the issue. They know that transplantation procedures save sick people's lives and are safe for living donors. They come into contact with serious illness and death on a daily basis, so they are aware of how much good such a procedure can do for many people (19, 22).

Majority of authors support the view that the role of the nurse in transplantation is very important. Most of them are positive and willing to participate in the organ donation process (23–25). Studies by other authors show that nurses' attitude and physicians' trust are the strongest predictors of consent (26). All agree that nurses are professionals who are in better contact with families than doctors and a study conducted in Spain indicates that nurses' attitudes are fundamental in the context of organ donation. Nurses with negative attitudes toward organ donation can influence those in contact with the subject or create distrust in the process (27). The treatment team tasked with helping patients and their families make the decision to donate organs at the end of life is of great importance in public education. Hence, their knowledge and attitude are important in planning to increase organ donation rates (28) and nurses’ low level of knowledge and negative attitude toward organ donation can exacerbate the lack of organ availability for transplantation. Therefore, interventions in the form of workshops, audiovisual presentations, and certification courses should be provided to nurses so that they can expand their knowledge of organ donation (10). In a German survey of nurses and students, they were asked if post-mortem organ donation was sufficiently covered in their university classes. 39.5% of medical students, 60.4% of health sciences students and 51.9% of nurses said they had not had classes on the subject (29).

In our own survey, more than 80.1% of respondents claimed they would be willing to donate organs after death to a stranger as well as a relative. In other surveys, nurses would accept someone else's organ in the event of a transplant need, although they do not have a donor card, but only a few would donate their organs. It is possible that the bias is due to ignorance, but in addition to negative attitudes, nurses expressed interest in learning and professional development in the field of transplantation (30). Another study by this author found that the mean knowledge score on organ donation was high. There was a statistically significant difference in scores between the two groups divided by seniority and level of education, which showed the strongest influence on nurses' attitudes toward donation. In addition, respondents did not express strong positive attitudes toward this issue. Further education of nurses in the health care system on organ and tissue transplantation and donation is warranted (31). In our own study, just under 2.0% of respondents do not support the idea of transplantation. The results of other authors show that the reason for the lack of acceptance of donating one's own organs after death is ignorance of medical problems and the lack of consent to organ donation was due, among other things, to the respondents' fear for their own health (15). Studies by other authors have shown that higher levels of knowledge about brain death are associated with greater support for transplantation (32).

In our own study, only 34.5% of the nurses surveyed took any action to promote the idea of transplantation, such as talking to the family of a potential donor. Studies by other authors in this area have shown that nurses lack knowledge about the concept of brain death, the legality of the donor and the donation process. Nurses also lacked the confidence and ability to initiate conversations with families about donation. They also recognized the need for more and ongoing education about organ donation (33). Nurses in one study noted that there is a lack of guidelines to support donation (34). Another study noted the need to raise awareness with the implementation of educational programs among health care professionals about organ donation and transplantation (35), since they are not only strategically positioned as the main intermediaries between organ donors and transplant recipients, but are also professionally involved in the process of organ donation and transplantation, and are often blamed for the global organ shortage (36).

Health care professionals' awareness and knowledge of transplant medicine can improve people's sensitivity and reduce their opposition to donation. The medical literature contains numerous examples of attitudes toward transplantation and organ donation directed at university students or medical staff, but rarely at nurses. Nurses are an important opinion-forming group in the patient population, and their negative attitudes can have a significant negative impact on public attitudes toward organ donation (6).

## 5. Conclusions

The conducted survey can be considered the basis for a wide-ranging study of the reasons for negative public attitudes toward organ donation. In Poland, nursing is a profession of public trust, so it is important to emphasize significant role of nurses in educating the public about organ procurement for transplantation.

The survey confirmed the thesis that the nurses surveyed are knowledgeable about transplantation and have a positive attitude toward organ donation and transplantation. The survey showed that, in the nurses' opinion, it is the deficit in the public's knowledge about the procurement of cells, tissues and organs from living donors and people who have been declared dead as a result of being pronounced dead that is the reason for opposition to organ transplantation. Therefore, it is extremely important to conduct extensive public campaigns and educational actions in this regard.

The study also found that there is a need for training, educational programs for nurses to enhance their role in organ donation and transplantation, especially courses in interpersonal communication to facilitate interviews with families of potential donors. Another solution could be to include transplantation in the nursing curriculum to ensure a general good knowledge of tasks related to this issue.

## 6. Limitations of the study

The survey was conducted among a group of nurses employed at health care facilities over a certain period of time, which may result in generalizable conclusions. By taking part in the survey, nurses provided subjective opinions and the current psychological well-being influenced the respondents' assessment of the situation. During the survey, there was an opportunity to exchange opinions among nurses.

## Data Availability

The raw data supporting the conclusions of this article will be made available by the authors, without undue reservation.
